# New Method of Treating Urethral Strictures

**Published:** 1877-03

**Authors:** 


New Method of Treating Urethral Strictures (*The Medical Record*, January 13, 1877).—At the meeting of the Académie de Médcine at Paris, on November 7, 1876, M. C. Fort described a method of treating urethral strictures of small calibre, which he has employed for seven years with great success. He first passes a filiform bougie through the stricture and leaves it in position for twenty-four hours. The presence of the bougie excites a slight inflammation of the tissues that form the stricture, which softens them and renders them more extensible. The bougie is provided with a metallic cap, to which catheters of various sizes may be attached. At the end of twenty-four hours a conical catheter, three millimetres in diameter at the largest part, is attached to the bougie and pushed forward through the stricture, the guide keeping it in the proper direction. The catheter is then withdrawn until the metallic cap on the end of the bougie emerges from the meatus. The catheter is now detatched from the bougie and replaced by another, which is five millimetres in diameter at its largest part. This is in turn made to traverse the stricture, then withdrawn, and replaced by a third catheter which measures seven millimetres in its largest diameter. The bougie during the passage of the catheters coils up in the bladder, where it can do no harm. By this method even very tight and indurated strictures may be completely dilated at a single sitting. The guide removes all danger of making a false passage. The pain is so slight that anæsthesia is unnecessary. The operation rarely causes any hemorrhage.—*Med. Times.*

